# Effect of Fibers Configuration and Thickness on Tensile Behavior of GFRP Laminates Exposed to Harsh Environment

**DOI:** 10.3390/polym11091401

**Published:** 2019-08-26

**Authors:** Milad Bazli, Hamed Ashrafi, Armin Jafari, Xiao-Ling Zhao, R.K. Singh Raman, Yu Bai

**Affiliations:** 1 Department of Civil Engineering, Monash University, Clayton, Victoria 3800, Australia; 2 Structural Research Center, International Institute of Earthquake Engineering and Seismology (IIEES), Tehran 19537-14453, Iran; 3 Department of Civil Engineering, Sharif University of Technology, Tehran 11365-11155, Iran; 4 Department of Mechanical and Aerospace Engineering, Monash University, Clayton, Victoria 3800, Australia; 5 Department of Chemical Engineering, Monash University, Clayton, Victoria 3800, Australia

**Keywords:** UV, moisture, freeze/thaw cycles, unidirectional fibers, woven fibers, random fibers, chopped strand fibers, durability

## Abstract

The present study indicates the importance of using glass fiber reinforced polymer (GFRP) laminates with appropriate thickness and fibers orientation when exposed to harsh environmental conditions. The effect of different environmental conditions on tensile properties of different GFRP laminates is investigated. Laminates were exposed to three environmental conditions: (1) Freeze/thaw cycles without the presence of moisture, (2) freeze/thaw cycles with the presence of moisture and (3) UV radiation and water vapor condensation cycles. The effect of fiber configuration and laminate thickness were investigated by considering three types of fiber arrangement: (1) Continuous unidirectional, (2) continuous woven and (3) chopped strand mat and two thicknesses (2 and 5 mm). Microstructure and tensile properties of the laminates after exposure to different periods of conditioning (0, 750, 1250 and 2000 h) were studied using SEM and tensile tests. Statistical analyses were used to quantify the obtained results and propose prediction models. The results showed that the condition comprising UV radiation and moisture condition was the most aggressive, while dry freeze/thaw environment was the least. Furthermore, the laminates with chopped strand mat and continuous unidirectional fibers respectively experienced the highest and the lowest reductions properties in all environmental conditions. The maximum reductions in tensile strength for chopped strand mat laminates were about 7%, 32%, and 42% in the dry freeze/thaw, wet freeze/thaw and UV with moisture environments, respectively. The corresponding decreases in the tensile strength for unidirectional laminates were negligible, 17% and 23%, whereas those for the woven laminates were and 7%, 24%, and 34%.

## 1. Introduction

Use of fiber-reinforced polymer (FRP) composites is rapidly increasing in automotive, aerospace, and marine applications, as well as in civil engineering [1,2,3]. These composites have several advantages, including lightness, high strength, ease of installation and handling, corrosion resistance, and relatively good durability properties, which have made them an attractive alternative to traditional materials (i.e., steel) in many applications [4,5,6]. The unique characteristics of FRPs also make them useful in engineering infrastructure for the rehabilitation and strengthening of existing structures [7,8]. However, despite their outstanding characteristics, FRPs also have disadvantages, such as low ductility, low shear strength (due to the weak mechanical performance of the resin), vulnerability to extreme temperatures and poor durability when exposed to alkaline conditions, ultraviolet (UV) radiation, freeze/thaw cycles or fluctuating hydrothermal conditions [9,10,11,12,13,14]. Consequently, the use of FRP composites in nonstructural elements, secondary structures, and for rehabilitation and strengthening of existing structures is limited, particularly, in situations where there are greater chances of exposure to the regular harsh environmental conditions, as well as to elevated temperatures and fires during structural service life, such as during transportation or storage [15,16].

The current literature on the degradation of FRP composites has examined the effects of different environmental factors, including alkaline and acidic solutions [17,18,19,20,21,22,23,24], seawater [17,19,21,24,25,26,27,28,29], hydrothermal conditions [30,31,32,33,34], temperature fluctuations [25,27,35,36,37,38], UV radiation [11,28,34,39,40,41,42,43], and elevated temperatures [11,39,44]. However, relatively fewer studies have focused on the performance of FRPs subjected to the harsh environmental conditions posed by the freeze/thaw cycles that simulate cold region climates or by the UV, moisture, and elevated temperature conditions that simulate sunny days in hot regions [12,45,46,47,48].

UV radiation is one of the factors responsible for the degradation of organic material, as it can break chemical bonds and initiate oxidation reactions. Although this degradation tends to be superficial and may not have any significant effect on mechanical properties of FRPs [11,49], a combination of UV radiation and moisture, together with elevated temperature, may intensify the adverse effects of these factors [39].

The potential damage resulting from diffusion of moisture into the polymer includes resin matrix cracking, swelling, plasticization, hydrolysis, and fiber/resin interface debonding, which are the primary causes of the degradation of the mechanical properties of composites [50,51].

Elevated temperature alone (without the presence of the moisture) is not a critical issue when it is below the glass transition temperature, *T*_g_, of the resin (which is 80 °C for the resin of FRP used in this study) [52,53]. The rapid degradation of FRPs due to elevated temperature starts when the temperature reaches the *T*_g_, when the resin matrix begins to change from a glassy to a rubbery state [54,55]. However, moisture penetration into the matrix may also be faster and deeper at elevated temperatures.

By contrast, matrix hardening (increased brittleness) and microcracks may occur when FRPs are exposed to subzero temperatures. The difference in the coefficients of thermal expansion between the resin and fibers in a composite material may also increase the internal stress in FRPs and lead to fiber/resin debonding in response to thermal variations. One conclusion, therefore, is that thermal cycles at low temperatures may be more damaging to the mechanical properties of FRPs when compared to constant exposure to freezing temperatures [56]. Further, the microcracks that can develop due to subzero temperatures may increase resin hydrolysis, plasticization, and moisture penetration into the resin matrix, thereby, further degrading the FRP mechanical properties. In other words, thermal cycling in the presence of moisture (wet cycling) can cause moisture to be absorbed and trapped in the resin cracks at higher temperatures, while subsequent freezing and expansion at low temperatures can cause crack growth and further resin/fiber debonding [47,57,58]. Studies on the effects of freeze/thaw cycles on the mechanical properties and degradation of FRP composites are limited [59]. Therefore, the present study investigates mechanical properties and microstructural degradation of glass fiber reinforced polymer (GFRP) composites, fabricated by vacuum infusion process, under freeze/thaw cycles (in the presence or absence of moisture).

Previous research into the effects of regular environmental factors (viz., UV radiation, moisture, and thermal fluctuations) on FRP composites has mostly focused on the effects of such factors on the structural behavior (e.g., bonding between the FRP laminate and the member substrate) of structures reinforced and/or strengthened with FRP [45,46,60,61,62,63,64]. Limited studies have also investigated the effects of parameters that influence the mechanical properties of the FRPs themselves after environmental exposures [37,39,65]. In addition, the literature related to the degradation of the mechanical properties of FRP composites subjected to harsh daily environmental conditions has mainly concentrated on only one type of fiber configuration (i.e., continuous-unidirectional fibers) [37,45,66]. However, other fiber characteristics, such as length and orientation, are also expected to affect the fabrication quality, mechanical properties and structural performance of FRPs [44,67], that, in turn, can affect the degradation mechanisms and penetration and propagation of cracks, and, consequently, the mechanical properties of the composite materials after exposure to harsh environmental conditions. To the best of the authors’ knowledge, the effect of fiber configuration on the durability and mechanical properties of FRP composites subjected to harsh environments remains poorly studied. Therefore, the effect of various fiber configurations is addressed in the present study by considering three types of GFRP composites: Unidirectionally orientated, woven orientated, and chopped strand (randomly orientated) fibers.

The behavior of FRP composites due to fiber orientation may be classified into two categories: (1) Anisotropic behavior for composites with fibers orientated in different directions, due to direct and shear-strain coupling effects [68] and (2) almost orthotropic behavior for composites with unidirectional fibers, due to their negligible strain coupling effects [69].

In addition to the fiber configuration, the thickness of the composites may play an important role in determining the damage resulting from environmental agents such as moisture, UV radiation and temperature fluctuations. It is known that the effects of moisture on composites are localized to a thin sub-surface layer that is exposed to moisture. Moreover, it is well established that UV radiation may mostly affect the surface (outer layer) of the composites and the inner layers are generally unaffected. Therefore, the effect of the exposed (damaged) layer will become more pronounced when a significant portion of the tested area is affected by the local damage zone: The thinner the sample, the greater is the chance of measuring the conditioning adverse effects [66]. However, based on the literature, the effect of specimen thickness on the mechanical properties of FRPs after exposure to harsh environmental conditions has received very limited attention [44]. Therefore, the present study will investigate this effect by considering different thicknesses of GFRP composites when exposing to environmental conditioning.

With the background described above, this experimental study investigates the effect of different parameters, i.e., the configuration of the fibers (length and orientation), specimen thickness, and number of conditioning cycles on mechanical and microstructural properties of GFRP composites after exposure to three environmental conditions: (1) UV radiation and water vapor condensation cycles, (2) freeze/thaw cycles without moisture, and (3) freeze/thaw cycles with moisture.

## 2. Experimental Description

The present study, as part of an ongoing research project on the durability of FRP composites used for structural applications, focuses on the tensile properties of GFRP laminates subjected to regular harsh environmental conditions. Thin laminates were tested in tension to simulate the condition of retrofitting structural members with FRP laminates. The flexural and compressive properties of FRP composites, when used as individual structural members under aggressive environments, will be studied by the authors in the future.

The effects of fiber length and orientation were investigated by considering unidirectional continuous, woven continuous, and chopped strands fibers. Moreover, the thickness effect was studied using two different thicknesses. Regarding the conditioning scheme, three regimes of cycling: (1) Sequential exposure of UV radiation and water vapor condensation, (2) freeze/thaw cycles with the presence of moisture (i.e., wet cycling) and (3) freeze/thaw cycles without the presence of moisture (i.e., dry cycling) were used. In order to study the effect of exposure time, three durations of cycling for each regime were used. Mechanical properties of the specimens after exposure to different periods of conditioning were investigated using tensile tests. Furthermore, scanning electron microscopy (SEM) analyses using TESCAN VEGA//XMU (TESCAN, Brno, Czechia) instrument was carried out after conditioning (both before and after mechanical tests) to study the microstructural degradation, damage progression, and failure modes. Finally, in order to quantify the obtained results and contribution of each variable on the final results, statistical study, including analysis of variance (ANOVA) and linear Bayesian regression were carried out.

### 2.1. Materials

**Figure 1:**
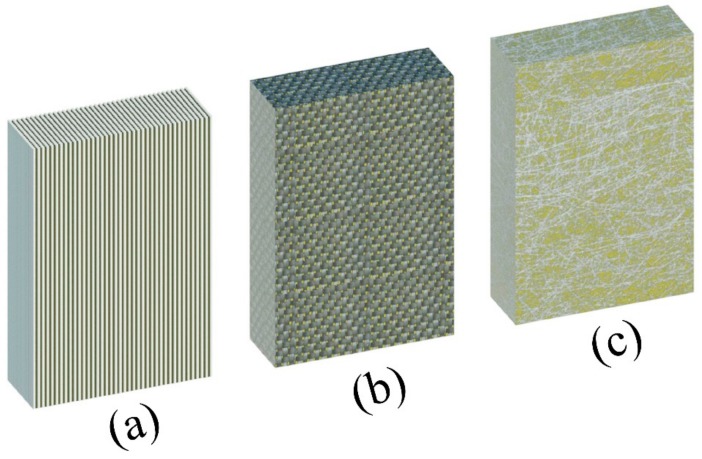
Different types of glass fiber reinforced polymer (GFRP) laminates used in this study: (**a**) Continuous unidirectional fibers, (**b**) continuous woven fibers and (**c**) chopped strand fibers.

Specimens were produced by vacuum infusion process (VIP). Details of VIP can be found in [44]. Continuous E-glass fibers were used for laminates with unidirectional and perpendicular (0° and 90°) woven fibers, while 25 ± 5 mm long E-glass fibers were used to produce randomly distributed laminates (chopped strand mat). The tensile strength, elastic modulus and density of E-glass fibers used were, 2900–3100 MPa, 70 GPa, and 2.6 g/cm^3^, respectively based on the manufacturer. IN2 epoxy infusion resin with tensile strength, viscosity, and density of about 65.5–73.5 MPa, 200–450 mPa·s and 1.08–1.12 g/mL (all at 20 °C) was used. All laminates were cured in room temperature for 24 h. Moreover, laminates were kept in 50 °C for 8 h to complete the post-curing cycle. Samples of 2- and 5-mm thicknesses were used. Figure 1 shows the samples used for this study. Table 1 lists the characteristics of laminates. Tensile tests were carried out by the authors to obtain the mechanical properties of the samples. The details of the test procedure are presented in Section 2.3 and Section 2.4.

### 2.2. Environmental Conditioning 

**Figure 2:**
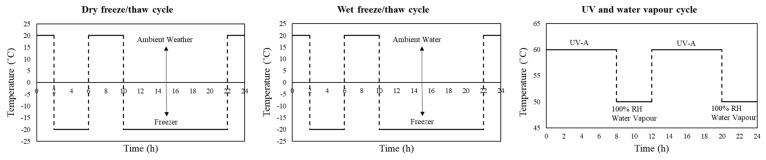
Environmental condition cycles used in this study.

Figure 2 shows the environmental condition schemes used in this study. From each sample type, three identical un-conditioned specimens were tested in tension and the average ultimate tensile strength was used as a reference for each condition. Samples were subjected to environmental conditioning for three durations: 750, 1250, and 2000 h.

It is worth mentioning that as there is no specification for the number of thermal cycles that FRPs may experience during their life-span, the number of conditioning cycles was estimated according to the standard recommendations for other non-structural and coating materials (i.e., EN 1504-4 [71] and ASTM D6944-15 [72]). In those standards, the maximum number of 50 cycles is recommended for a life span of 25 years. Accordingly, the maximum cycling duration of 2000 h was chosen in this study, which is a reasonable estimation for about 30 to 50 years of service life under very cold conditions.

As the first environmental conditioning, the samples were exposed to the cycles of UV radiation and water vapor condensation according to the standard, ASTM G154 [73]. As shown in Figure 2, each cycle constituted 8 h of UV–A radiation at 60 °C followed by 4 h of water vapor condensation at 50 °C with 100 % RH. A UV–A lamp with the radiation′ wavelength of 340 nm, and an irradiate of 0.85 W/m^2^ was used to simulate a regular harsh environment of coastal areas [74].

For each dry freeze/thaw cycle, which lasted for 24 h, the samples were held for 12 h at a sub-zero temperature (−20 °C), followed by 4 h at the ambient temperature (20 °C), 4 h of subzero temperature (as a shock to maximize the conditioning effect), and finally, 4 h of ambient temperature.

For wet freeze/thaw cycling, the samples were held for 14 h at a subzero temperature (−20 °C), followed by 8 h of immersion in tap water at 20 °C. It is important to note that the temperature range (−20 to 20 °C) was selected according to the recommendations [36] for testing to simulate very cold regions (e.g., those in Canada).

### 2.3. Specimens

A total of 204 laminates were prepared for conditioning and mechanical tests. The length and width of all samples were 300 and 60 mm, respectively. To examine the reproducibility of data, test in each condition was triplicated. In order to prevent possible failure and slip at the ends of the sample (grip of the testing machine), two tabs were attached to the tensile samples before conducting the tests. It should be noted that tabs were attached after conditioning.

### 2.4. Tensile Tests 

**Figure 3:**
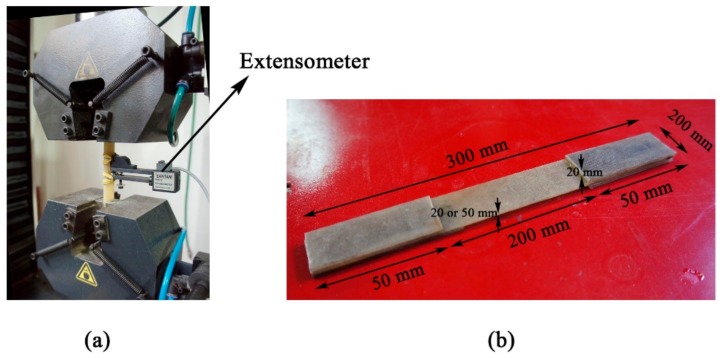
Tensile test: (**a**) Test set-up and (**b**) sample configuration.

Tensile properties of control and conditioned GFRP laminates were determined by tensile tests using a Santam (Tehran, Iran) servo electrical testing machine. All tensile samples were subjected to increasing load at a constant loading rate of 1.2 mm/min, until fracture. Strain was measured using an extensometer attached at the middle of the sample. Figure 3 shows the tensile test set-up and the sample configuration.

### 2.5. Scanning Electron Microscopy (SEM) 

In order to investigate the degradation mechanisms of the resin matrix, fibers, and their interface as well as the damage propagation (e.g., cracks configuration and fiber/matrix debonding) under different conditioning, SEM analyses were conducted on a few randomly selected samples after exposure to different freeze/thaw cycles and before mechanical tests. SEM was also carried out on the samples that were fractured in tensile tests.

## 3. Results and Discussion

Degradation in GFRP and their mechanical properties, and the degradation mechanisms are discussed in this section.

### 3.1. Microstructural Changes due to Conditioning

**Figure 4:**
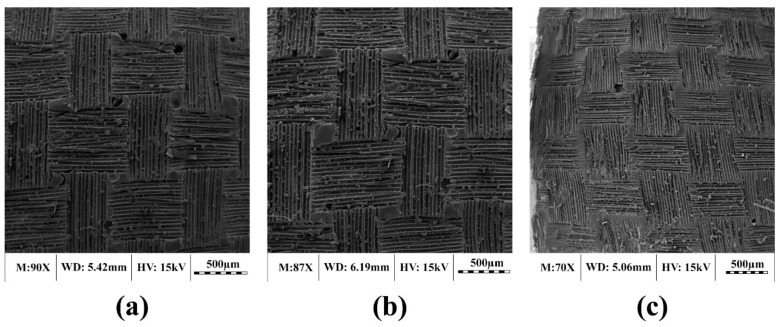
SEM micrographs of control laminates: (**a**) Unidirectional laminate (UL), (**b**) woven laminate (WL) and (**c**) chopped strand laminate (RL).

To investigate the degradation mechanism of GFRP composites, the surfaces of selected laminate samples before (control) and after (conditioning) exposure to different environmental conditions were observed under SEM. Figure 4 shows the control samples of each laminate type used in this study. It is noted that, in order to achieve a relatively rough surface during fabrication that was required for gripping the tensile test samples, the manufacturer used a layer of perpendicular woven fiber on the sample surface. However, this layer was removed at the final stage. Therefore, as seen in Figure 4, all studied laminate types have a final woven shaped resin matrix layer (i.e., the uppermost layer does not comprise woven fibers). Therefore, all laminates, as seen in Figure 3, have just a woven shaped matrix surface (there is no woven fabric). As expected, no considerable damage (e.g., significant cracks in the resin matrix) is observed in the control samples. The holes and cracks seen in the resin matrix are attributed to sample fabrication, handling or loading.

**Figure 5:**
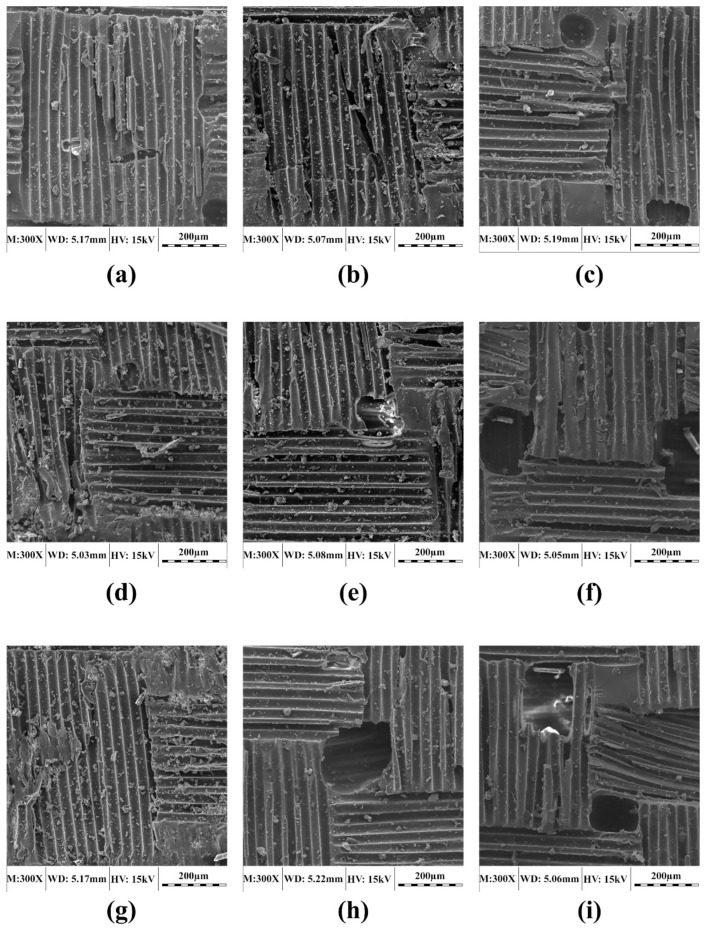
SEM micrographs of selected 2-mm laminates exposed to dry freeze/thaw cycles: (**a**) UL exposed to 750-h cycles, (**b**) UL exposed to 1250-h cycles, (**c**) UL exposed to 2000-h cycles, (**d**) WL exposed to 750-h cycles, (**e**) WL exposed to 1250-h cycles, (**f**) WL exposed to 2000-h cycles, (**g**) RL exposed to 750-h cycles, (**h**) RL exposed to 1250-h cycles and (**i**) RL exposed to 2000-h cycles.

Figure 5 shows SEM images of the selected laminates exposed to dry freeze/thaw cycles, indicating that a greater number of holes and cracks is produced with an increasing number of cycles. These cracks and holes in the resin matrix may be attributed to the matrix degradation as well as the differences in the thermal expansion coefficients of fiber and resin. However, considerable fiber/resin debonding was not observed in the examined samples. It should be mentioned that UL, WL, and RL represent unidirectional, woven and chopped strand mat laminates, respectively.

**Figure 6:**
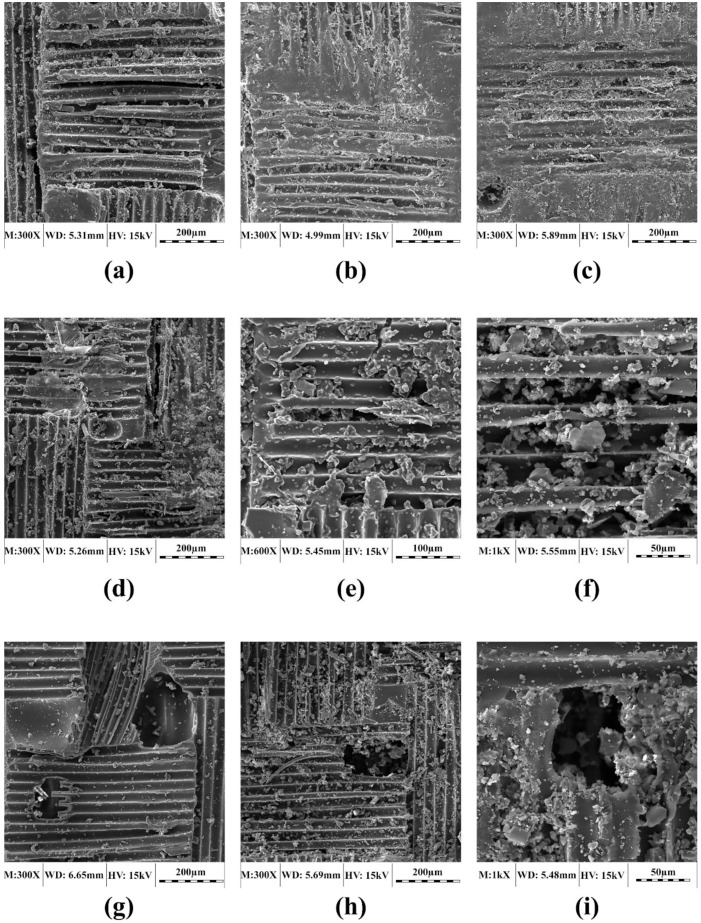
SEM micrographs of selected 2-mm laminates exposed to wet freeze/thaw cycles: (**a**) UL exposed to 750-h cycles, (**b**) UL exposed to 1250-h cycles, (**c**) UL exposed to 2000-h cycles, (**d**) WL exposed to 750-h cycles, (**e**) WL exposed to 1250-h cycles, (**f**) WL exposed to 2000-h cycles, (**g**) RL exposed to 750-h cycles, (**h**) RL exposed to 1250-h cycles and (**i**) RL exposed to 2000-h cycles.

Figure 6 shows SEM images of the selected laminates exposed to wet freeze/thaw cycles. It is also evident that increasing the number of cycles in the wet freeze/thaw condition may cause greater damage to resin matrix, leading to fiber/matrix debonding that may degrade the composite′s mechanical properties. Extensive fiber/resin debonding is seen for laminates subjected to 2000 h of wet freeze/thaw cycles. Obviously, the damage incurred in the wet cycles is more severe than that resulting from dry cycles, which is attributed to the additional deterioration of the resin matrix due to the absorbed moisture in the wet cycle.

**Figure 7:**
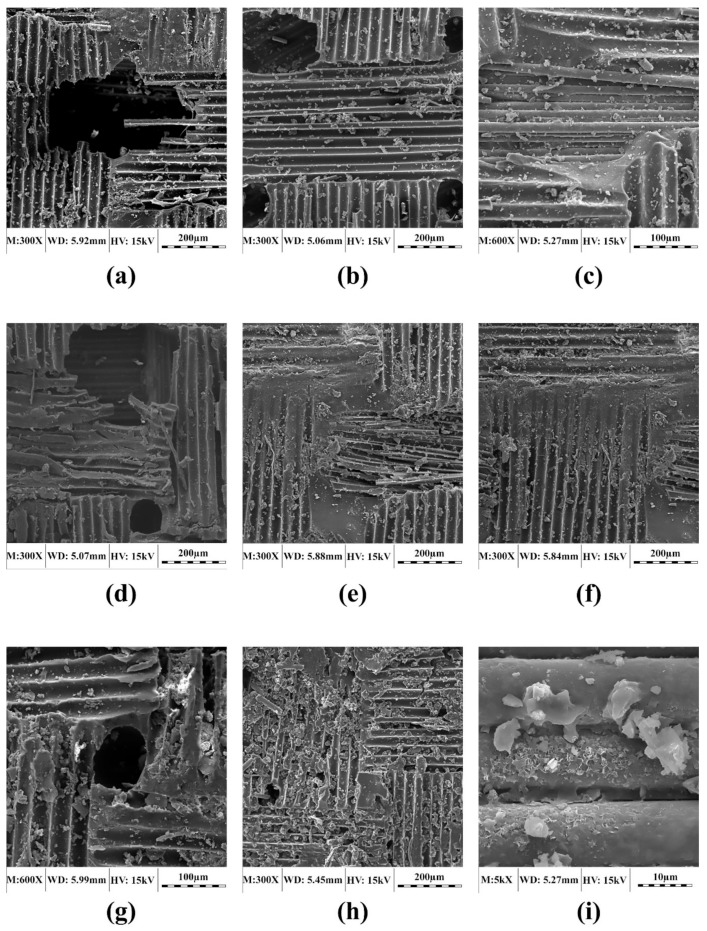
SEM micrographs of selected 2-mm laminates exposed to UV and water vapor condensation cycles: (**a**) UL exposed to 750-h cycles, (**b**) UL exposed to 1250-h cycles, (**c**) UL exposed to 2000-h cycles, (**d**) WL exposed to 750-h cycles, (**e**) WL exposed to 1250-h cycles, (**f**) WL exposed to 2000-h cycles, (**g**) RL exposed to 750-h cycles, (**h**) RL exposed to 1250-h cycles and (**i**) RL exposed to 2000-h cycles.

Figure 7 shows SEM images of the selected laminates exposed to UV radiation and water vapor condensation cycles that caused more pronounced cracks and holes in the resin matrix and fiber/resin debonding than the freeze/thaw cycles, indicating that UV radiation and moisture cycles represent the harshest environment. From Figure 6, it is also evident that, after exposure to 2000 h of conditioning cycles, the surface resin layer was severely damaged and the fibers were obviously exposed and debonded. Therefore, in addition to possible swelling, plasticization and cracking due to moisture, chemical changes in the resin matrix due to the processes involving UV radiation and oxygen can be distinguished as the main factors contributing to such significant deterioration of the laminates. The greater damages in the UV radiation and moisture cycles has a direct bearing on the tensile test properties (as will be demonstrated later).

### 3.2. Failure Modes

**Figure 8:**
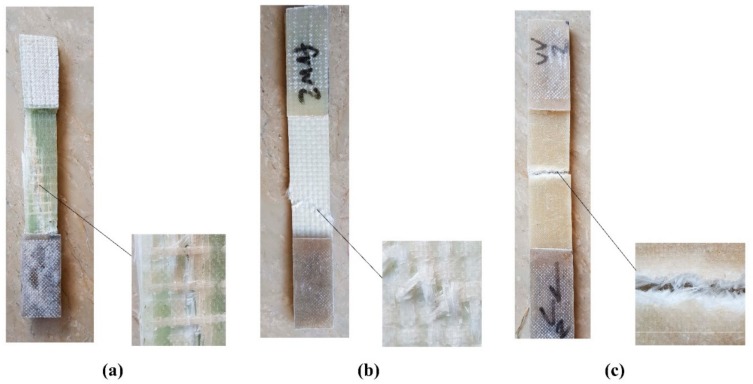
Typical failure modes of GFRP laminates: (**a**) Unidirectional laminate, (**b**) woven laminate and (**c**) chopped strand laminate.

It is noteworthy that a similar failure mode was observed in all conditioned and control samples of each laminate type. In other words, environmental conditions did not affect the failure modes of the control samples. Based on the ASTM D 3039/D 3039M [70] standard, three failure modes were observed during the tensile tests. SEM images were also taken from the fracture surface of some selected samples for a close inspection of the failure modes. Figure 8a shows the typical failure mode of the unidirectional GFRP laminate. UL samples failed due to the long splitting of the fibers in the gauge length of the sample. Figure 8b shows the typical failure mode of the woven GFRP laminate. WL specimens failed between the gauge length of the laminates following the zigzag (angled) pattern of weaving. This failure included fracturing of the fibers orientated in the applied load direction, as well as deformation and delamination of the fibers orientated perpendicular to the applied load direction Figure 8c shows the typical failure mode of the chopped strand mat GFRP laminate. In the case of RL samples, since a significant part of the applied load is carried by the resin (a limited number of the fibers are orientated in the same direction as the load applies), both resin matrix and the fibers failed through a lateral failure at a section within the gauge length of the laminates.

### 3.3. Tensile Test Results

The elastic modulus results of conditioned samples showed no significant changes compared to the control samples. In other words, the results for all samples exposed to different conditions were within the standard deviation of the control samples. The possible reason for this observation is an insignificant degradation of fibers, as the main factor in tensile elastic modulus value of FRP, due to the environments. Therefore, by considering the observation that the tensile elastic modulus of GFRP laminates is not affected by the UV radiation and freeze/thaw cycles, the tensile strength variation of such laminates will be studied in the following sections.

Table 2 presents the tensile test results for the control and conditioned GFRP laminates. For sample identification, a four-part notation system was used, whereby the first letter represents the fiber type, namely, U for unidirectional, W for woven, and R for random. The second and third letters denote sample thickness and time of exposure, respectively, and the last letter stands for the environmental condition, whereas FD and FW represent dry freeze/thaw and wet freeze/thaw cycles, respectively, while UV denotes the cycles of ultraviolet radiation and water vapor moisture. For example, U-2-1000-UV shows the 2-mm GFRP laminate with unidirectional fibers exposed to 1000 h of UV and water vapor cycles.

In order to investigate the effect of each parameter, first, the results for each fiber configuration will be studied, and then all laminate types will be compared with each other to find out their relative performance under different environmental conditions.

#### 3.3.1. Unidirectional GFRP Laminates (UL)

**Figure 9:**
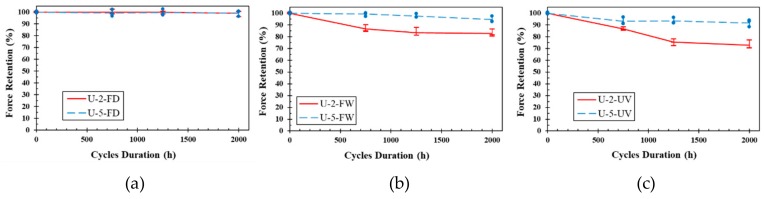
Retention ultimate tensile strength vs. exposure period of UL: (**a**) Dry freeze/thaw cycles, (**b**) wet freeze/thaw cycles and (**c**) UV and water vapor condensation cycles.

Figure 9 shows the retention in ultimate tensile strength vs. exposure period of unidirectional fiber laminates. It is seen that the environment of UV and moisture cycles was the most aggressive environment while dry free/thaw condition was the least damaging. The maximum reduction of UL samples was about 27% related to U-2-2000-UV. No significant change was observed for UL specimens subjected to FD (up to 1%). This shows that unidirectional GFRP laminates are resistant to freeze/thaw cycles, even without the presence of moisture. Greater reduction in tensile strength of GFRP laminates under FW cycles compared to that in FD (up to 8% for U-2-2000-FW) suggests the damaging role of moisture in degradation of FRPs. The greater resistance of the thicker samples to environmental damage may be simply due to the lesser fraction of sample thickness getting penetrated by moisture and getting damaged. For the environment of UV and moisture, it is well known that UV radiation mostly affects the surface of the composites [39]. Hence, the greater fraction of sample thickness will be getting damaged in the thinner laminates compared to the thicker laminates.

**Figure 10:**
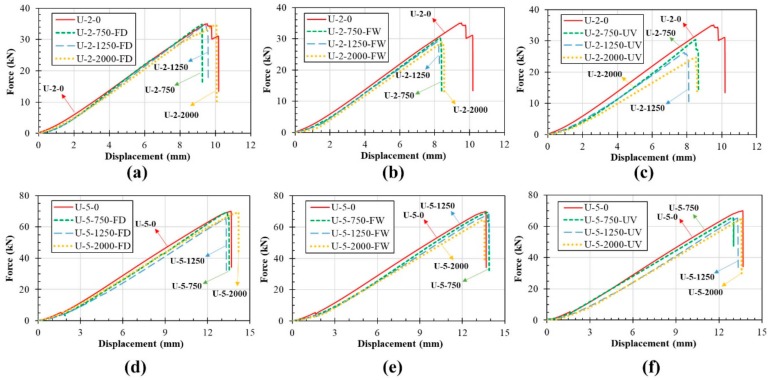
Load-deflection curve of 2-mm UL laminates: (**a**) 2-mm laminate exposed to dry freeze/thaw (FD), (**b**) 2-mm laminate exposed to wet freeze/thaw (FW), (**c**) 2-mm laminate exposed to UV, (**d**) 5-mm laminate exposed to FD, (**e**) 5-mm laminate exposed to FW and (**f**) 5-mm laminate exposed to UV.

Figure 10 shows the load vs. deflection curves of the control and conditioned UL laminates. As expected, FRP laminates under tensile loading showed a viscoelastic behavior up to the failure.

#### 3.3.2. Woven GFRP Laminates (WL)

**Figure 11:**
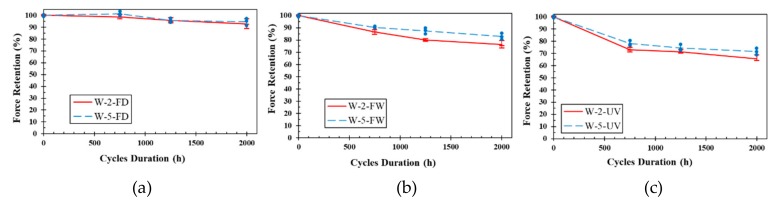
Retention ultimate tensile strength vs. exposure period of WL: (**a**) Dry freeze/thaw cycles, (**b**) wet freeze/thaw cycles and (**c**) UV and water vapor condensation cycles.

Figure 11 shows the retention in ultimate tensile strength vs. exposure period of woven fiber laminates. Similar to UL specimens, WL specimens experienced the highest reductions under UV and moisture environment (the maximum reduction of UV and moisture environment was 35% for U-2-2000-UV) and the lowest reductions under dry freeze thaw environment (the maximum reduction of dry freeze thaw environment was 7% for U-2-2000-FD). WL samples exposed to wet freeze/thaw cycles experienced a maximum of 24% reduction in tensile strength (Table 2). This significant difference between the reductions in the dry and wet freeze/thaw cycles confirms that the presence of moisture may considerably enhance the adverse effect of freeze/thaw cycles on the mechanical properties of FRPs. In addition to the adverse effects of moisture on composites [50,51], freezing and expanding the moisture inside the laminates, which may cause crack growth and resin/fiber debonding [57,58] may be another reason for this difference. Less reduction in properties of the 5-mm samples compared to the 2-mm samples is consistent with the similar findings for UL laminates (Section 3.3.2).

**Figure 12:**
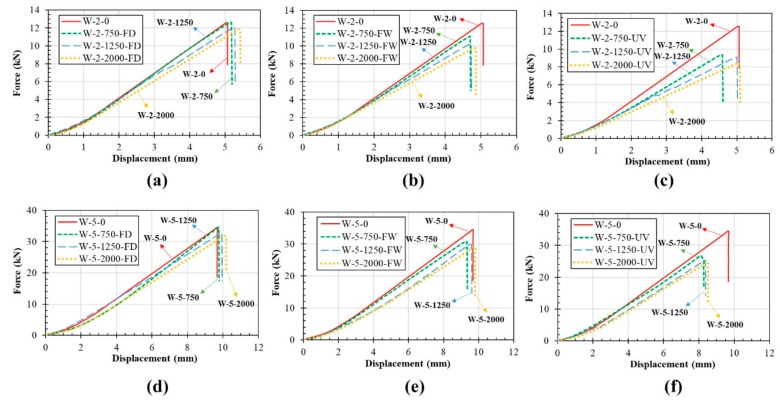
Load-deflection curve of WL: (**a**) 2-mm laminate exposed to FD, (**b**) 2-mm laminate exposed to FW, (**c**) 2-mm laminate exposed to UV, (**d**) 5-mm laminate exposed to FD, (**e**) 5-mm laminate exposed to FW and (**f**) 5-mm laminate exposed to UV.

Figure 12 shows the load vs. deflection curves of the control and conditioned WL laminates. Similar to UL samples, a linear elastic behavior was observed for all control and conditioned WL samples.

#### 3.3.3. Chopped Strand Mat GFRP Laminates (RL)

**Figure 13:**
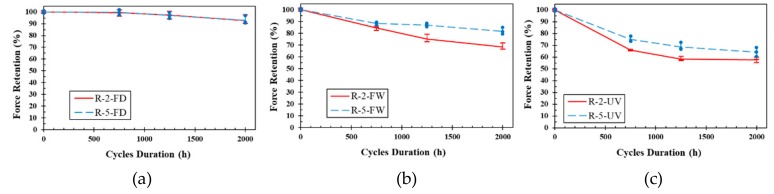
Retention ultimate tensile strength vs. exposure period of RL: (**a**) Dry freeze/thaw cycles, (**b**) wet freeze/thaw cycles and (**c**) UV and water vapor condensation cycles.

Figure 13 shows the retention in ultimate tensile strength vs. exposure period of random fiber laminates. The performance of RL samples was similar to that of UL and WL in terms of the degradation rates in different environmental conditions as well as the effect of thickness. The maximum degradation of RL laminates in each environmental condition was about 42%, 32%, and 7% for R-2-2000- UV, R-2-2000-FW, and R-2-2000-FD, respectively. Considerable difference in strength reductions was observed for thicker laminates compared to the thinner ones under wet freeze/thaw and UV with moisture conditions. However, no significant difference was seen under dry freeze/thaw cycles. The maximum reductions for 5-mm laminates were about 36%, 19%, and 7% for R-5-2000- UV, R-5-2000-FW, and R-5-2000-FD, respectively. This observation reveals that the moisture penetration and UV radiation effects are strongly related to the thickness, while the adverse effect of dry freeze/thaw cycles may not change considerably with changing in the sample thickness. This was also confirmed for UL and WL samples (Table 2).

**Figure 14:**
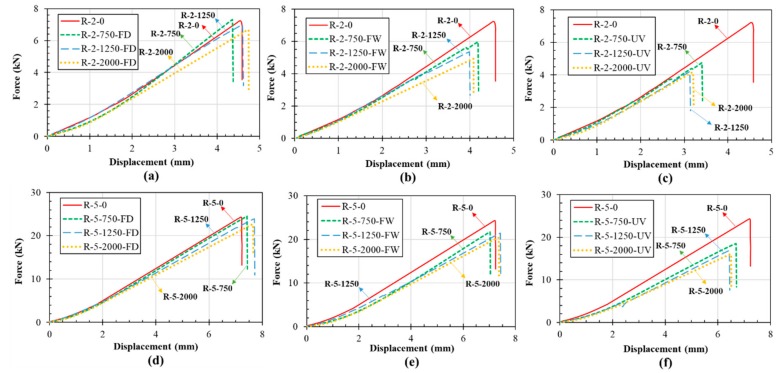
Load-deflection curve of RL: (**a**) 2-mm laminate exposed to FD, (**b**) 2-mm laminate exposed to FW and (**c**) 2-mm laminate exposed to UV, (**d**) 5-mm laminate exposed to FD, (**e**) 5-mm laminate exposed to FW and (**f**) 5-mm laminate exposed to UV.

Figure 14 shows the load vs. deflection curves of the control and conditioned RL laminates. Although the behavior of RL samples, in terms of linear elastic behavior, was the same as UL and WL, the RL samples were failed with a significantly lower sound than UL and WL specimens.

#### 3.3.4. Comparison between Different Types of GFRP Laminates 

**Figure 15:**
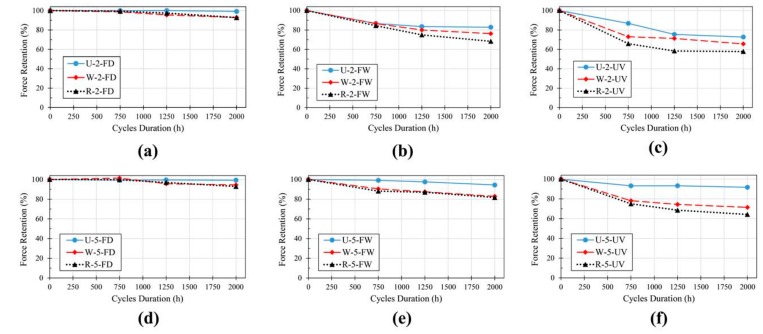
Comparison between the tensile strength reductions of GFRP laminates: (**a**) 2-mm laminate exposed to FD, (**b**) 2-mm laminate exposed to FW, (**c**) 2-mm laminate exposed to UV, (**d**) 5-mm laminate exposed to FD, (**e**) 5-mm laminate exposed to FW and (**f**) 5-mm laminate exposed to UV.

Figure 15 compares the retention in ultimate tensile stress vs. exposure period of different GFRP laminates exposed to different environmental conditioning. Among the three laminate types, generally, UL samples showed the best performance, while RL samples showed the weakest performance under different environmental conditions. In general, it can be concluded that, regardless of the type of laminates, GFRP laminates used in this study were resistant to freeze and thaw cycles without the presence of moisture (maximum reduction of 7%). The other two conditions were detrimental due to the presence of moisture, which could intensify the adverse effects of freeze/thaw and UV cycles. It is well known that the resin matrix degradation is the primary factor responsible for the degradation of FRPs’ mechanical properties [75]. Therefore, the laminates with continuous unidirectional fibers are resistant to material degradation as they have more fibers in the direction of the applied load compared to the woven and random fiber laminates. This explanation is also valid for the comparison of WL and RL. As the mechanical properties of RL are more dependent on the properties of resin matrix than WL (since more fibers are orientated in the direction of applied loads in WL than RL), the reductions in tensile strength of RL are higher than WL laminates.

#### 3.3.5. Comparison the Results with Similar Studies

In this section, the results of the present study are compared with those of similar studies reported by other researchers. Table 3 and Table 4 summarize the results of this study and some similar studies under freeze/thaw cycles and UV with moisture cycles conditions, respectively.

From Table 3 and the results reported by Wu et al. [59] on flexural properties of 3.5-mm plates from a GFRP vinylester resin deck system, which was made out of two layers of fabric (each layer included fibers orientated in 0°, 90°, 45°, −45°, and random directions) subjected to freeze/thaw cycles in air and water, it can be concluded that generally, FRP composites are resistant to freeze/thaw cycles without the presence of the moisture. Wu et al. [59] also found freeze/thaw cycles carried out in the temperature range of 4.4 to −17.8 °C in water do not have a significant difference in loss in mechanical properties as compared to the FRPs conditioned in distilled water.

As is seen in Table 4, the results for unidirectional FRP laminates, regardless of the fabrication method, are comparable. In addition, the reductions observed for woven and chopped strand GFRP laminates were greater than the results reported for all unidirectional laminates. This comparison confirms the greater susceptibility of the FRP composites with fewer fibers along the applied load, i.e., the lesser the fibers in the same direction of the applied load, the higher is the strength reductions. In other words, the tensile properties of laminates with woven and chopped strand fibers are governed more by the resin properties than those of unidirectional laminates, and thus, laminates with woven and chopped strand fibers are more vulnerable under environmental conditions.

## 4. Statistical Analyses

### 4.1. Analysis of Variance (ANOVA) 

To investigate the contribution percentage of each variable used in this study, namely the type of the laminates, conditioning period and sample thickness in tensile strength reduction of GFRP laminates after exposure to environmental conditions, two-way ANOVA analyses were used. Two sets of analyses were carried out to investigate all variable effects. First, the type of laminates considered as the constant factor and exposing period and laminate thickness considered as variables. Next, the thickness of the laminates was considered as the constant factor and type of the laminate and exposing period were considered as the analyses’ variables.

The details of the procedure for ANOVA analyses can be found in [81]. Significantly high error for UL under dry freeze/thaw environment was obtained, which confirms that the laminates are less dependent on the tests variables and, as shown in tensile strength results, resistant to the dry freeze/thaw condition. The results for both wet freeze/thaw and UV with moisture conditions revealed that the testing variable, namely thickness and exposing periods, are significantly effective factors in tensile strength reductions of unidirectional GFRP laminates. However, the effect of thickness is higher in wet freeze/thaw condition than that of UV and moisture condition (46% compared to 32% contribution).

For woven and chopped strand laminates, as expected, the conditioning period is the most important factor with more than 80% contribution for all conditions. Similar to UL samples, the effect of thickness is higher under wet freeze/thaw cycles than UV and water vapor cycles (8% contribution compared to 2% for woven laminates and 13% contribution to 4% for chopped strand laminates).

For all conditions, the effect of exposing period is the main factor with the minimum of 53% contribution on the final tensile retention results. The exposing time has the lowest and highest effects on 2-mm laminates under dry freeze/thaw and wet freeze/thaw conditions, respectively (53%, 87%, and 82% for dry freeze/thaw, wet freeze/thaw and UV with moisture conditions, respectively). The possible reason for the low dependency of the dry freeze/thaw condition to the exposing time may be due to the less adverse effect of freeze/thaw cycles on the laminates in terms of tensile strength. However, the effect of exposing period in 5-mm laminates decreased for other two conditions (55%, 55%, and 53% for dry freeze/thaw, wet freeze/thaw, and UV with moisture conditions, respectively). This observation may confirm the fact that due to the lower ratio of the damaged layers to the testing area for 5-mm laminates compared to 2-mm laminates, the rate of degradation is smaller for thicker laminates with respect to the exposure time.

### 4.2. Fitting Models 

According to the tensile test results and ANOVA analyses, probability models were proposed using linear Bayesian regression models with the help of risk tools (RT) software [82]. Thickness and time of exposure were considered as the effective parameters on tensile strength retentions. To confirm the validity of proposed models, various regression parameters, including the regression coefficient, homoscedasticity, non-collinearity, and error normality were checked for numerous models. Finally, Equation (1) was selected to predict the tensile strength retention of unidirectional, woven and chopped strand mat GFRP laminates after exposure to dry freeze/thaw cycles, wet freeze/thaw cycles, and UV radiation and water vapor cycles conditions. It should be mentioned that, due to the large error value in ANOVA analyses of UL samples exposed to FD condition (i.e., no reductions observed for UL sample in that condition), the proposed formula does not predict the values of UL in FD condition.

(1) $$R\;\left(\%\right)=A\;\left(\frac{1}{Log\left(T\right)}\right)+B\;\left(T^{0.33}\right)+c$$

where ***T*** (h) is the exposure period, ***t*** (mm) is the laminate thickness; and A, B, and C are constants based on each type of GFRP laminates. Table 5 summarizes the equations’ constants for each environmental condition.

**Figure 16:**
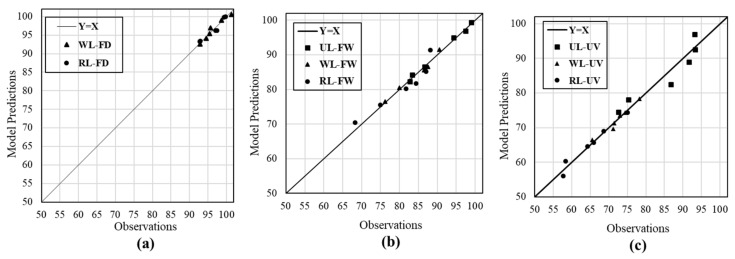
Comparison between the test results and model predictions: (**a**) FD, (**b**) FW, and (**c**) UV.

Statistical parameters of linear Bayesian regression for each probability model is presented in Table 6. According to the results, the reliability of the equations is confirmed. However, using more experimental data to improve the accuracy of the proposed models with considering different possible effective variables, such as materials’ characteristics and condition temperature is recommended. The results of tensile strength obtained from the experimental tests with the results calculated based on the fitted models for each environmental condition were compared in Figure 16. As it is seen, an acceptable agreement is achieved for the predictions, which confirms the high accuracy of the proposed models.

## 5. Conclusions

This study was conducted as part of a research program related to the degradation of FRP composites under environmental conditions. Future studies will focus on the durability of FRP composites when used for civil engineering applications (e.g., using with seawater sea sand concrete). In the present study, the mechanical and microstructural properties of unidirectional, woven, and chopped strand mat GFRP laminates were studied using tensile tests and SEM analyses. The following conclusions were drawn based on the observations and analyses:

(1) The tensile elastic modulus of the GFRP laminates is not considerably affected by the UV radiation and freeze/thaw cycles.

(2) In all environmental conditions unidirectional and chopped strand mat GFRP laminates showed the best and the worst tensile performance, respectively, while the woven GFRP laminates performed between those two. This may be due to the amount of fibers, which are the greatest for unidirectional and the lowest for the strand laminates, in the direction of the applied load. The greater the amount of the fibers in the longitudinal direction, the less the degradation of tensile mechanical properties.

(3) Based on SEM analyses, cracks and holes in resin matrix increase by the exposure time. Accordingly, due to the damage growth, considerable resin/fiber interface debonding was observed when the conditioning period reached 2000 h for most of the samples exposed to different conditions.

(4) All three types of GFRP laminates are almost resistant to the freeze/thaw cycles without the presence of the moisture (maximum reduction was about 7%).

(5) Presence of moisture in freeze/thaw cycles increases the damage level of GFRP laminates due to the freezing and expanding the absorbed moisture, which leads to the crack growth and fiber/matrix debonding (e.g., maximum reduction of 32% for random fiber laminate subjected to wet freeze/thaw compared to 7% for dry freeze/thaw).

(6) UV radiation and water vapour condensation condition are found to be the most aggressive environment among all three environmental conditions. Maximum reductions of about 27%, 34% and 42% were found, after 2000 h exposing, for 2-mm unidirectional, woven, and chopped strand mat GFRP laminates, respectively.

(7) The sample thickness is an important factor in the tensile performance of laminates exposed to daily environmental conditioning. This is due to the fact the daily conditions mainly affect the surface of the composites rather than the inner layers. The maximum difference in tensile strength reduction about 19%, 6%, and 13% were observed between 2- and 5-mm laminates after exposure to different environmental conditions for unidirectional, woven, and chopped strand mat samples, respectively.

As this study shows, several factors, such as the materials characteristics and fabrication method, composites cross-section (i.e., thickness), exposing time may affect the degradation level of FRP composites under daily environmental conditions. Therefore, conducting more experimental tests in order to obtain more data to better understand the performance of FRPs under such conditions and possible modelling of this influence is recommended.

## Figures and Tables

**Table 1:** Characteristics of GFRP laminates constructed by vacuum infusion process (VIP) method.

Property	U		W		R	
Thickness (mm)	2	5	2	5	2	5
Number of layers	6	16	6	17	3	7
Fiber to resin ratio by volume (fiber (%)/resin (%))	70.5/29.5	71.2/28.8	65.2/34.8	64.6/35.4	41.6/59.4	33.1/66.9
Arial weight of each roving layer (gr/m^2^)	350	350	350	350	350	
Tensile strength of single roving layer based on manufacturer data-sheet (MPa)	750–921		297–342		negligible	
Thickness of single roving layer based on manufacturer data-sheet (mm)	0.27		0.25		0.53	
Roving elongation based on manufacturer data-sheet (%)	3		2		Not-specified	
Count of yarns (threads/cm)	4.5		4 (Wrap) × 4 (Fill)		Not-specified	
Ultimate tensile strength (MPa) (mean ± standard deviation)	700 ± 5.6	872 ± 5.3	320 ± 1.8	341 ± 2.5	180 ± 2.2	246 ± 2.8
Tensile elastic modulus (GPa) (mean ± standard deviation)	32 ± 1.1	33 ± 0.8	18 ^a^ ± 0.1, 11^b^ ± 0.1	22.0 ^a^ ± 0.2, 15 ^b^ ± 0.1	11.0 ± 0.1	15 ± 0.2
Glass transition temperature, *T*_g_ (°C)	80	80	80	80	80	80
Thermal expansion coefficient (10^−6^/°C)	0.3–0.6	0.3–0.6	0.3–0.6	0.3–0.6	0.3–0.6	0.3–0.6

Note: U = unidirectional, W = woven, R = random, W laminates have bilinear stress-strain curve [70]: (a) Initial elastic modulus, (b) secondary chord elastic modulus.

**Table 2:** Test results.

Specimen	Average Ultimate Stress (MPa)	CV (%)	Retention (%)	Specimen	Average Ultimate Stress (MPa)	CV (%)	Retention (%)	Specimen	Average Ultimate Stress (MPa)	CV (%)	Retention (%)
U-2-0-FD	872.2	0.61	100.0	W-2-0-FD	320.1	0.57	100.0	R-2-0-FD	180.1	1.24	100.0
U-2-750-FD	871.5	2.39	99.9	W-2-750-FD	316.0	1.88	98.7	R-2-750-FD	178.9	2.67	99.3
U-2-1250-FD	872.0	1.29	100.0	W-2-1250-FD	305.9	2.46	95.5	R-2-1250-FD	175.4	3.37	97.4
U-2-2000-FD	864.8	2.65	99.2	W-2-2000-FD	297.3	4.32	92.9	R-2-2000-FD	167.1	4.68	92.8
U-2-0-FW	872.0	0.61	100.0	W-2-0-FW	320.1	0.57	100.0	R-2-0-FW	180.1	1.24	100.0
U-2-750-FW	756.0	3.47	86.7	W-2-750-FW	277.0	2.12	86.5	R-2-750-FW	152.0	2.21	84.4
U-2-1250-FW	728.0	4.48	83.5	W-2-1250-FW	256.0	1.33	80.0	R-2-1250-FW	135.0	4.85	75.0
U-2-2000-FW	722.0	3.96	82.8	W-2-2000-FW	244.0	3.97	76.2	R-2-2000-FW	123.0	4.89	68.3
U-2-0-UV	872.2	0.61	100.0	W-2-0-UV	320.1	0.57	100.0	R-2-0-UV	180.1	1.24	100.0
U-2-750-UV	756.6	1.84	86.8	W-2-750-UV	233.7	2.44	73.0	R-2-750-UV	118.6	1.02	65.9
U-2-1250-UV	657.5	3.63	75.4	W-2-1250-UV	228.0	2.01	71.2	R-2-1250-UV	105.0	3.67	58.3
U-2-2000-UV	634.2	5.15	72.7	W-2-2000-UV	210.0	4.17	65.6	R-2-2000-UV	104.0	4.23	57.7
U-5-0-FD	700.1	0.81	100.0	W-5-0-FD	341.6	0.72	100.0	R-5-0-FD	246.0	1.14	100.0
U-5-750-FD	695.6	2.90	99.3	W-5-750-FD	346.4	1.85	101.4	R-5-750-FD	245.3	2.09	99.7
U-5-1250-FD	696.9	2.71	99.5	W-5-1250-FD	326.8	1.97	95.7	R-5-1250-FD	238.5	2.55	97.0
U-5-2000-FD	694.6	2.54	99.2	W-5-2000-FD	322.7	3.13	94.5	R-5-2000-FD	228.5	3.61	92.9
U-5-0-FW	700.1	0.81	100.0	W-5-0-FW	341.6	0.72	100.0	R-5-0-FW	246.0	1.14	100.0
U-5-750-FW	694.0	1.54	99.1	W-5-750-FW	309.0	0.94	90.5	R-5-750-FW	217.0	1.54	88.2
U-5-1250-FW	683.0	1.88	97.6	W-5-1250-FW	299.0	2.81	87.5	R-5-1250-FW	214.0	1.84	87.0
U-5-2000-FW	661.0	2.72	94.4	W-5-2000-FW	283.0	3.07	82.8	R-5-2000-FW	201.0	3.57	81.7
U-5-0-UV	700.1	0.81	100.0	W-5-0-UV	341.6	0.72	100.0	R-5-0-UV	246.0	1.14	100.0
U-5-750-UV	652.2	3.48	93.2	W-5-750-UV	267.0	2.58	78.2	R-5-750-UV	184.6	3.35	75.1
U-5-1250-UV	653.1	2.95	93.3	W-5-1250-UV	254.0	3.55	74.4	R-5-1250-UV	168.7	4.71	68.6
U-5-2000-UV	642.0	3.29	91.7	W-5-2000-UV	244.0	3.73	71.4	R-5-2000-UV	158.0	5.85	64.2

Note: CV = coefficient of variation.

**Table 3:** Test results of the present study and other research under freeze/thaw cycles.

Reference	FRP Type	Environmental Condition	Results
Present study	Vacuum infusion GFRP epoxy laminates	Dry freeze/thaw cycles from −20 to 20 °C	negligible, 7% and 7% reductions for 2-mm unidirectional, woven and chopped strand mat laminates, respectively after 2000-h cycles
Present study	Vacuum infusion GFRP epoxy laminates	Wet freeze/thaw cycles from −20 to 20 °C	17%, 24%, and 32% reductions for 2-mm unidirectional, woven and chopped strand mat laminates, respectively after 2000-h cycles
[57]	Wet lay-up GFRP vinylester laminates.	Dry freeze/thaw cycles from −10 to 22.5 °C	9.1% reduction for laminates with three layers of 0.125-mm stabilized unidirectional fabric after 2400-h cycles
[57]	Wet lay-up GFRP vinylester laminates.	Wet freeze/thaw cycles (in deionized water) from −10 to 22.5 °C	9.6% reduction for laminates with three layers of 0.125-mm stabilized unidirectional fabric after 2400-h cycles
[76]	Wet lay-up GFRP epoxy laminates.	Dry freeze/thaw cycles from −30 to 30 °C	No significant change in tensile strength after 90 cycles
[47]	pultruded GFRP isophthalic polyester sheet	Dry freeze/thaw cycles from −10 to 20 °C	No significant change in tensile strength after 300 cycles
[47]	pultruded GFRP isophthalic polyester sheet	Wet freeze/thaw cycles from −10 to 20 °C	13% reduction for 6.4-mm laminates with five roving layers (three continuous strand mats and two unidirectional rovings) after 300 cycles

**Table 4:** Test results of the present study and other research under UV radiation and moisture environment.

Reference	FRP Type	Environmental Condition	Results
Present study	Vacuum infusion GFRP epoxy laminates	UV radiation and water vapor condensation cycles	27%, 34%, and 42% reductions for 2-mm unidirectional, woven and chopped strand mat laminates, respectively after 2000-h cycles
[77]	Pultruded GFRP vinylester laminates	UV radiation and water vapor condensation cycles	25% reduction for 2-mm laminates after 2000-h cycles
[78]	hand lay-up flax fabric reinforced epoxy composites	UV radiation and water spray cycles	29% reduction of 5.2-mm laminate after exposure to 1500-h cycles
[79]	Pultruded GFRP polyester/vinyl ester laminates	Combined effect of high temperature, freeze–thaw cycles, moisture and UV radiation	15.5%, 10.4%, and 13.9% reduction for 2.85-mm isophthalic polyester, orthophthalic polyester, and vinyl ester laminates after six months
[80]	Pultruded GFRP polyester laminates	UV radiation and moisture cycles	21% reduction of 5-mm laminates after exposure to 3000-h cycles

**Table 5:** Constant values in Equation (1) for FD condition.

Specimen Type	Environmental Condition	A	B	C
UL	FD	-	-	-
WL	FD	142.988	3.337	45.088
RL	FD	145.893	0.074	49.056
UL	FW	95.744	28.258	17.664
WL	FW	200.12	13.424	−0.959
RL	FW	250.179	21.657	−32.576
**UL**	UV	175.939	32.114	−19.254
**WL**	UV	1156.327	10.531	5.930
**RL**	UV	215.628	19.283	−33.639

**Table 6:** Bayesian regression parameters for FD condition.

Specimen Type	Environmental Condition	R-Factor	Mean of Standard Deviation
UL	FD	-	-
WL	FD	0.98	0.949
RL	FD	0.97	0.954
UL	FW	0.99	0.737
WL	FW	0.99	1.062
RL	FW	0.96	3.063
UL	UV	0.94	4.12
WL	UV	0.98	1.09
RL	UV	0.99	1.62
